# A Mechanistic Tumor Penetration Model to Guide Antibody Drug Conjugate Design

**DOI:** 10.1371/journal.pone.0118977

**Published:** 2015-03-18

**Authors:** Christina Vasalou, Gabriel Helmlinger, Bruce Gomes

**Affiliations:** Advanced Quantitative Sciences, Novartis, Cambridge, MA, United States of America; University of Catania, ITALY

## Abstract

Antibody drug conjugates (ADCs) represent novel anti-cancer modalities engineered to specifically target and kill tumor cells expressing corresponding antigens. Due to their large size and their complex kinetics, these therapeutic agents often face heterogeneous distributions in tumors, leading to large untargeted regions that escape therapy. We present a modeling framework which includes the systemic distribution, vascular permeability, interstitial transport, as well as binding and payload release kinetics of ADC-therapeutic agents in mouse xenografts. We focused, in particular, on receptor dynamics such as endocytic trafficking mechanisms within cancer cells, to simulate their impact on tumor mass shrinkage upon ADC administration. Our model identified undesirable tumor properties that can impair ADC tissue homogeneity, further compromising ADC success, and explored ADC design optimization scenarios to counteract upon such unfavorable intrinsic tumor tissue attributes. We further demonstrated the profound impact of cytotoxic payload release mechanisms and the role of bystander killing effects on tumor shrinkage. This model platform affords a customizable simulation environment which can aid with experimental data interpretation and the design of ADC therapeutic treatments.

## Introduction

Antibody-Drug Conjugates (ADCs) are therapeutic hybrid constructs comprised of a potent cancer therapeutic molecule joined by a chemical linker to an antibody directed against a tumor surface antigen. The idea of using ADCs for the direct delivery of a cytotoxic agent to the target cells was first described in the 1980s. Recent development and commercialization of two ADCs, trastuzumab DM1 (TDM1) and brentuximab vedotin have demonstrated the practicality of this biotherapeutic modality. TDM1’s clinical success in breast cancer [1, 2] and brentuximab vedotin’s attainment of 35% complete and 40% partial remission in Hodgkin lymphoma [3] have heralded the beginning of a wave of clinical successes. There are currently more than 200 registered clinical trials for ADC reagents, in more than 50 diseases [4]. However, not all ADC compounds are successful. Gemtuzumab ozogamicin, (marketed as Mylotarg and used for the treatment of acute myelogenous leukemia) was removed from the market due an increased risk of veno-occlusive disease, leading to liver toxicity [5]. These setbacks have highlighted the complexity of designing a successful ADC therapy.

An ADC comprises three components, each having a distinct role: a) the antibody is designed to recognize specific tumor-associated antigens, b) the linker is designed to release cytotoxic payload within the cell, and c) the payload, usually a small molecular weight cytotoxic agent, achieves killing of the cancer cell. The success of an ADC therapy relies on each of these three components. Extensive optimization of these parameters has taken place over the past decades, resulting in a library of potential linker technologies and cytotoxic molecules that can produce pharmacologically active ADCs [6]. Despite this drive to maximize ADC therapeutic potential, mechanistic processes involved in the localization and activation of ADC components, combined with multiple design parameters, may result in non-intuitive drug distribution and effects, which would be difficult to understand without the use of a more systematic mathematical model.

Previous modeling efforts have explored the distribution of various biologics modalities within tumor tissues, focusing on the competing processes of target tissue binding *versus* diffusive transport [7, 8]. More recent work has focused on the biodistribution of monoclonal antibodies in a variety of preclinical species and human using more detailed physiologically-based pharmacokinetic models [9, 10]. Shah *et al*. further used a mechanistic PK/PD model to assess ADC and payload PK in various animal species, in order to predict clinical response in cancer patients, using brentuximab vedotin as an example [11]. In the present work, and for the first time, we present a mechanism-based PK/PD model that incorporates detailed descriptions of receptor dynamics, payload internalization and release mechanisms, to describe ADC and payload penetration at the cellular level and within a solid tumor, in order to project the effect in tumor mass fluctuations. Our model, currently parameterized for a mouse xenograft with the potential of extension to human, describes the importance of key intrinsic parameters, such as antigen (receptor) expression level and endocytic kinetics, in the selection of targets for ADCs; it also shows the influence of properties of the reagents that can be selected to increase the chance of a successful compound. This work presents a generic model to evaluate ADC tumor penetration that can be further calibrated to match tumor- and ADC-specific properties.

## Materials and Methods

The model components, reflective of the fundamental steps involved in tumor localization, included: 1) blood flow 2) extravasation, interstitial transport and local binding/target kinetics, as well as 3) tumor growth dynamics. The model assumed a Krogh cylinder geometry to describe drug distribution from a cylindrical blood vessel segment towards surrounding tissues.

### ADC Pharmacokinetics

The blood concentration was defined as a two-compartment model with a bi-exponential decay, characteristic of antibody pharmacokinetics. Typically, the local blood concentration within the Krogh cylinder is dependent on blood velocity, intravascular permeability, and the fraction of drug not bound to blood cells. Antibodies, however, due to their large size, demonstrate limited extravasation rates and are less susceptible to changes in blood flow. Concentration differences along the length of blood vessels can therefore be disregarded [7]. Blood concentration (*C*_*plasma*_) is described as follows: C_plasma_ = C_plasma,o_ [A*e^−ka*t^ + B*e^−kb*t^]*C*_*plasma*,*o*_ is the initial plasma concentration, *A* and *B* are the fractions and ka and kb are the clearance rates for the alpha and beta phases, respectively. ADCs were assumed to follow typical antibody PK, characterized by an initial, rapidly declining distribution phase (alpha phase) during which the drug transports from the plasma to surrounding tissues, followed by an elimination phase (beta phase) during which the drug gets cleared from the organism. A plasma volume of 2 ml and a weight of 20 g were assumed.

### Tissue Distribution and Target Receptor Kinetics

**Fig 1:**
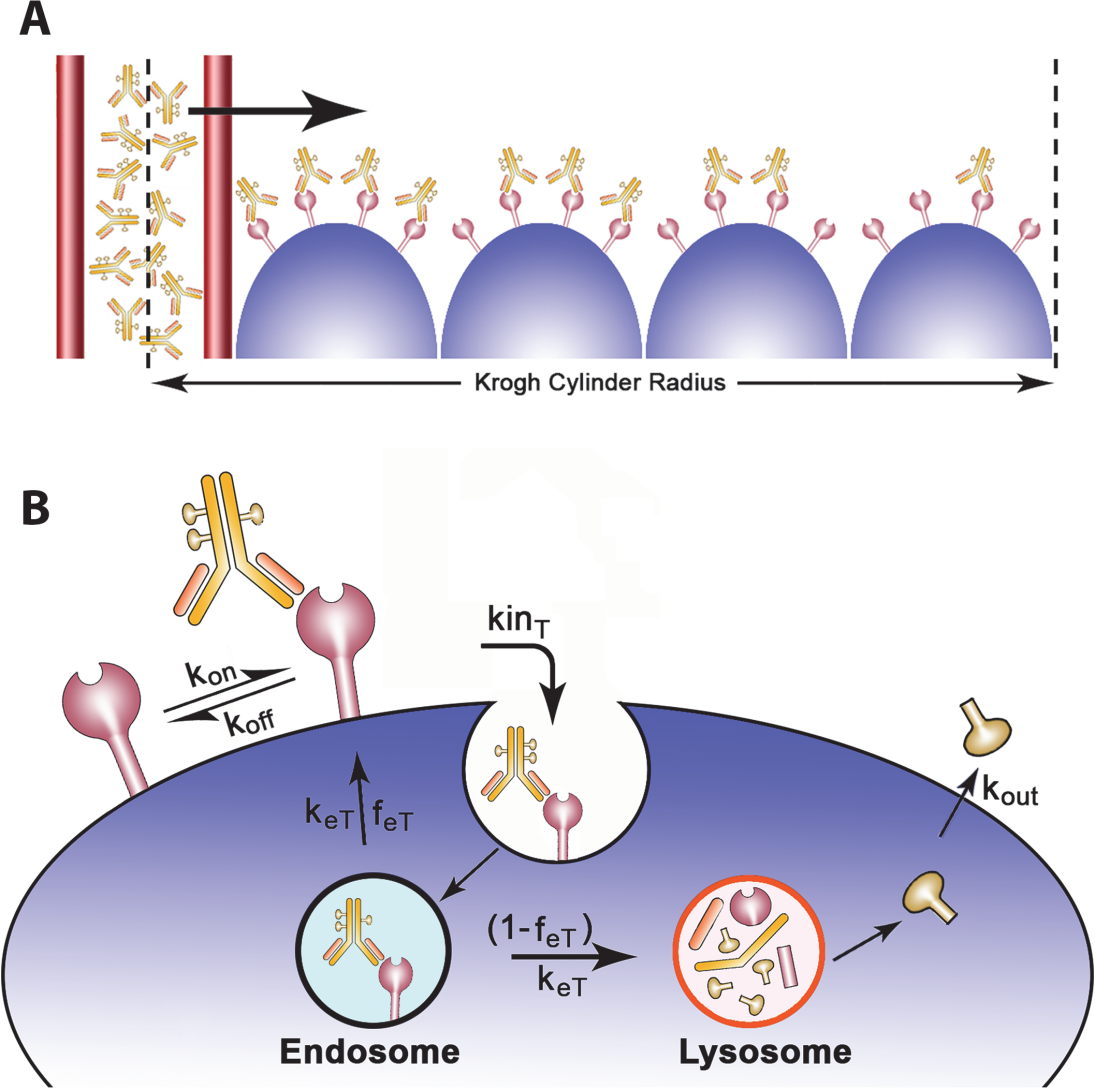
ADC tumor penetration schematic. A) A cross section of the Krogh cylinder illustrates the passage of ADCs through the capillary wall into the tumor tissue. The Krogh cylinder radius is equal to half the mean intercapillary distance, which is a tumor specific value. B) ADCs reversibly bind to antigen receptors located on the surface of tumor cells. Upon its formation, the ADC/receptor complex internalizes at a kinT rate and is further sorted in the endosomes. The complex exits the endosomes with a keT rate and is either recycled back to the surface with a feT fraction or degraded in the lysosomes. The model assumes payload release in the lysosomes in its nominal case; upon its release, the payload can diffuse out of the cytosol with a kout rate. The model assumes no payload reentry (no bystander killing effects) unless otherwise noted.

The tissue distribution model originates from a study published by Thurber *et al*. [7]. Tissue transport was governed by diffusion, hypothesized to occur through a homogeneous tissue. Model equations included only radial drug transport. Angular coordinates were disregarded under the assumption of axisymmetric drug penetration. Axial coordinates were also ignored, as previous work has demonstrated minimal variations along the length of the capillary segment due to the slower extravasation rates of antibodies [7]. The tissue distribution model therefore reduced to a one-dimensional problem, which can be visualized as a cross-section of the Krogh cylinder (Fig. 1A **).** Convective forces were not included in the model; previous studies have demonstrated that lack of functional lymphatic drainage elevates interstitial pressure, thereby reducing convective transport [12–14].

ADC distribution and receptor kinetics within the tumor tissue were described by the following equations:

$$\frac{d[C]}{dt}\;=\;D\;[\frac{1}{r}\frac{\partial \left[C\right]}{\partial r}+\frac{\partial^{2}[C]}{{\partial r}^{2}}]-Rxn$$ 1

$$\frac{d[T]}{dt}\;=\;Rs-Rxn-\;{kin}_{T}[T]+k_{eT}\;f_{eT\;}[T_{e}]$$ 2

$$\frac{d[B]}{dt}\;=\;Rxn-\;{kin}_{B}[B]+\;k_{eB}\;f_{eB\;}[B_{e}]$$ 3

$$\frac{d[T_{e}]}{dt}\;=\;{kin}_{T}\left[T\right]-k_{eT}\;f_{eT\;}[T_{e}]-k_{eT}\;{(1-f}_{eT\;})[T_{e}]$$ 4

$$\frac{d[B_{e}]}{dt}\;=\;{kin}_{B}\left[B\right]-\;k_{eB}\;f_{eB\;}\left[B_{e}\right]-\;k_{eB}\;{(1-f}_{eB\;})\left[B_{e}\right]$$ 5

Simplifying:

$$\frac{d[T_{e}]}{dt}\;=\;{kin}_{T}∙\left[T\right]-k_{eT}\;[T_{e}]$$ 6

$$\frac{d[B_{e}]}{dt}\;=\;{kin}_{B}\left[B\right]-\;k_{eB}\left[B_{e}\right]$$ 7

$$Rxn\;=\;k_{on}\frac{\left[C\right]}{\varepsilon }\left[\frac{\left[T\right]∙}{\varepsilon }\Gamma \right]-k_{off}\left[B\right]$$ 8

The receptor concentration at t = 0 (*T*_*init*_) was computed as:

$$T_{in}\;=\;\frac{\#Rec}{A_{v}\;V_{cell}}$$ 9

Where *#Rec* is the number of receptors per cell, *A*_*v*_ the Avogadro number, and *V*_*cell*_ the volume of the cell equal to *4/3π(R*_*d*_ *)* ^*3*^, *R*_*d*_ being the cell radius. The model assumes target receptor localization exclusively within the tumor tissue; receptor expression in healthy tissue, followed by target mediated drug disposition, has therefore not been explored in the context of this work.

Antibody (*C*) was transported through the tissue with an effective diffusion coefficient *D* and reversibly bound the target receptor (*T*) with an on/off rate, *k*_*on*_ and *k*_*off*_, respectively. Note that *k*_*off*_ = *k*_*on*_ * K_D_, K_D_ being the antibody dissociation constant. Antibody (*C*) and target (*T*) tissue concentrations are restricted to the extracellular tissue volume, which results in higher interstitial *versus* overall drug concentration. This increase in concentration is accounted for by dividing the concentration values by the void fraction (*ε*). Target concentration (*T*) is additionally multiplied with tumor cell mass Γ to account for the dynamic change in tumor mass, directly influencing the target receptor concentration within the Krogh cylinder.

The endocytic trafficking dynamics model, obtained from a previous study by Hendriks *et al*. [15], described the endocytic internalization of the target receptor and the competing processes of lysosomal degradation *versus* recycling back to the cell surface (Fig. 1B **)**. Target receptors were synthesized at rate *Rs*, internalized at *kin*_*T*_ rate and exited endosomes at rate *k*_*eT*,_ with fraction *f*_*eT*_ recycling back to the surface and fraction (1- *f*_*eT*_) being degraded in the lysosomes. Antibody-receptor complexes (*B*) internalized at rate *kin*_*B*_ and were further sorted through in endosomes with an exit rate *k*_*eB*_, a recycling fraction *f*_*eB*_ and degradation fraction (1- *f*_*eB*_). *T*_*e*_ and *B*_*e*_ describe the concentrations of the target and the antibody-target complex, respectively, within the endosome.

$$\frac{d[C_{p}]}{dt}\;=\;k_{eB}\;{(1-f}_{eB\;})\left[B_{e}\right]*DAR-k_{out}\left[C_{p}\right]+k_{in}\frac{\left[C_{p,ext}\right]}{\varepsilon }$$ 10

$$\frac{d[C_{p,ext}]}{dt}\;=\;k_{out}\left[C_{p}\right]-k_{in}\frac{\left[C_{p,ext}\right]}{\varepsilon }+D_{p}\left[\;\frac{1}{r}\frac{\partial \left[C_{p,ext}\right]}{\partial r}+\frac{\partial^{2}\left[C_{p,ext}\right]}{{\partial r}^{2}}\right]$$ 11

The intracellular payload concentration *C*_*p*_ was released in the cytosol as a result of lysosomal degradation. We have therefore assumed non-cleavable linkers for the purpose of this model (endosomal degradation via cleavable linkers has also been explored in this study). Upon linker cleavage, the payload could further exit the cell at a *k*_*out*_ rate or reenter it at a *k*_*in*_ rate. *K*_*out*_ is a function of the retention half-life of the payload within the cell (*t*_*p*_):

$$k_{out\;}\;=\;\frac{ln\left(2\right)}{t_{p}}$$ 12

Upon its exit from the cytosol to the tumor interstitial space, payload *C*_*p*,*ext*_ diffuses with *D*_*p*_ at its effective diffusion coefficient. For the purpose of our simulations (and with the exception of our last figure), we assumed that the payload could not reenter tumor cells (*k*_*in*_ = 0, no bystander killing effect), descriptive of many commercially available payloads such as monomethyl auristatin E (MMAE).

The boundary condition at the intercapillary interface r = R_cap_ defined a diffusive flux equal to the flux at the capillary wall, as driven by permeability *P*. Vascular permeability is an essential model parameter which determines the amount of ADC passing through the capillary wall and entering the tumor tissue. The boundary condition at r = R_Krogh_ assumed no flux out of the cylinder.

$$D{\left.\frac{\partial [C]}{\partial r}\right|}_{r\;=\;R_{cap}}\;=\;P\left(\left[C_{plasma}\right]-\frac{\left[C\right]}{\varepsilon }\right)$$ 13

$${\left.\frac{\partial [C]}{\partial r}\right|}_{r\;=\;R_{Krogh}}\;=\;0$$ 14

### Tumor Mass Dynamics

Tumor mass dynamics equations were adjusted from Panetta *et al*. [16]. The model takes into consideration effects on the cell cycle and doubling time, as well as on the percentage of proliferating (*i*.*e*., sensitive to treatment) *versus* quiescent cells. The payload concentration had a direct effect on proliferating cells, as long as the IC_50_ value (defined as the payload concentration required to achieve a 50% reduction in cell mass at a pre-specified point in time) was exceeded.

$$\frac{dP}{dt}\;=\;\left(\gamma -\;\alpha -\;c_{p}∙E_{50}\right)P\;+\;\beta \;Q$$ 15

$$\frac{dQ}{dt}\;=\;\alpha P-\;\beta \;Q$$ 16

The proliferating cell mass (*P*) and quiescent cell mass (*Q*) depended on the relative growth of cycling cells (*γ*)—defined as growth rate *minus* natural cell decay, the transition rate from proliferating to resting (*α*), the transition rate from resting to proliferating (β), and the effect of payload accumulation in the cytosol. The term *E*_*50*_ ensured payload efficacy for *C*_*p*_ ≥ IC_50_.

$$E_{50}\;=\;\frac{{\frac{C_{p}}{\Gamma }}^{h}}{{\frac{C_{p}}{\Gamma }}^{h}+{IC50}^{h}}$$ 17

Where *h* is the Hill coefficient. The payload concentration *C*_*p*_ was normalized by *Γ* to correct for fluctuations in tumor cell mass.

The parameters *α* and *γ* can be defined further by solving the following set of equations:

$$Q\left(t_{d}\right)+P\left(t_{d}\right)\;=\;2\;(P\left(0\right)+Q(0))$$ 18

$$\frac{P}{Q}\;=\;r_{t}$$ 19

Where *t*_*d*_ is the doubling time and *r*_*t*_ the proliferating-to-quiescent cell mass ratio.

Solving the equations yields:

$$\gamma \;=\;\frac{\ln\left(2\right)}{t_{d\;∙\;}r_{t}}(r_{t}+1)$$ 20

$$\alpha \;=\;\frac{\ln\left(2\right)}{t_{d\;∙\;}r_{t}}+\frac{\beta }{r_{t}}$$ 21

Γ= P + Q 22

The tumor cell mass, *Γ*, was obtained by the addition of proliferating and quiescent cell masses. Γ at time zero was set equal to unity (the sum of proliferating and quiescent fractions). This mathematical manipulation enables the expression of tumor mass (*Γ*) for t>0 as a percent change from its initial value at time zero. As a reminder, the precise tumor size is not a required input to the model since we are using Krogh cylinder geometry. Tumor mass reduction is therefore normalized to its starting value.

All model parameters and relevant references can be found in Table 1. As previously mentioned, this work presents a generic tumor model and the chosen parameters reflect average values obtained from various literature reports. The model however can be calibrated to fit specific biological systems. To simulate a particular tumor type, for example, parameters such as the doubling time (*t*_*d*_), proliferating-to-quiescent cell ratio, Krogh cylinder radius (*RKrogh*), number of receptors per cell (*#Rec*), would need to be appropriately adjusted. The model was solved using the method of finite differences with a discretization step of 1 μm and was simulated using Matlab (The Mathworks; Natick, MA) with the SBPOP toolbox (www.sbtoolbox2.org), which significantly decreased simulation times.

**Table 1:** Model parameters used in simulations.

Symbol	Name	Nominal Values	Notes & References
A	Fraction of alpha clearance	60 (40–70%)	[8, 17]
B	Fraction of beta clearance	40 (30–60%)	[8, 17, 18]
ka	Clearance rate for alpha phase	0.6 (0.2–0.6) hr^−1^	[8, 18]
kb	Clearance rate for beta phase	0.17 (0.1–0.2) day^−1^	[7, 8, 18, 19]
R_Krogh_	Krogh cylinder radius	72 (30–200+) μm	[20–22]
R_cap_	Capillary radius	8 (5–15μm)	[20, 21, 23]
P	Permeability	2.8 ∙ 10^−7^ (1.1–5.3 ∙10^−7^) cm/s	[24, 25]
D	Effective diffusivity	1.3∙10^−7^ (0.5^−^1.9 ∙10^−7^) cm^2^/s	[8, 13, 26, 27]
k_on_	Forward reaction rate	6*10^-3 nM^−1^ min^−1^	[28, 29]
K_D_	Dissociation constant	0.1 (0.001–100) nM	Ab design parameter
ε	Void fraction	0.24 (0.17–0.36)	[8, 30, 31]
kin_T_	Target Receptor internalization rate	0.01 (10^−4^–0.5) min^−1^	[15, 29, 32, 33]
k_eT_	Target Receptor endosomal exit rate	0.03 (0.01–0.07) min^−1^	[15, 32]
f_eT_	Target Receptor recycling fraction	0.5 (0–0.95)	Dependent on selected target receptor [15]
kin_B_	Ab-Target complex internalization rate	0.01 (10^−4^–0.5) min^−1^	[15, 29, 32, 33]
k_eB_	Ab-Target complex endosomal exit rate	0.03 (0.01–0.07) min^−1^	[15, 32]
f_eB_	Ab-Target complex recycling fraction	0.5 (0–0.95)	Dependent on selected target receptor(15]
t_p_	Payload retention half-life	7 (16–22) hr	[34, 35]
k_in_	Payload re-entry rate into cytosol	0	No bystander effect assumed
DAR	Drug-Antibody ratio	3	
D_p_	Effective diffusion coefficient of payload	3.2∙ 10^−6^ cm^2^/s	[8]
β	Transition rate from resting to proliferating	0.05 days	[16]
t_d_	Doubling time	10 (5–20+) days	Dependent on xenograft[16, 36]
r_t_	Proliferating-to-quiescent cell mass ratio	0.10.9 (0.050.95–0.40.6)	Dependent on xenograft [16, 36]
IC50	IC_50_ concentration as measured experimentally	3 (0.1–10) nM	Dependent on type of target
h	Hill coefficient	100	
#Rec	Number of receptors per cell	10^5^ (10^4^–2∙10^6^)	Dependent on type of target [15]
Rcell	Cell radius	8 (5–12) μm	[37–39]

## Results

### Intrinsic (System) Properties

#### Target Receptor Properties

**Fig 2:**
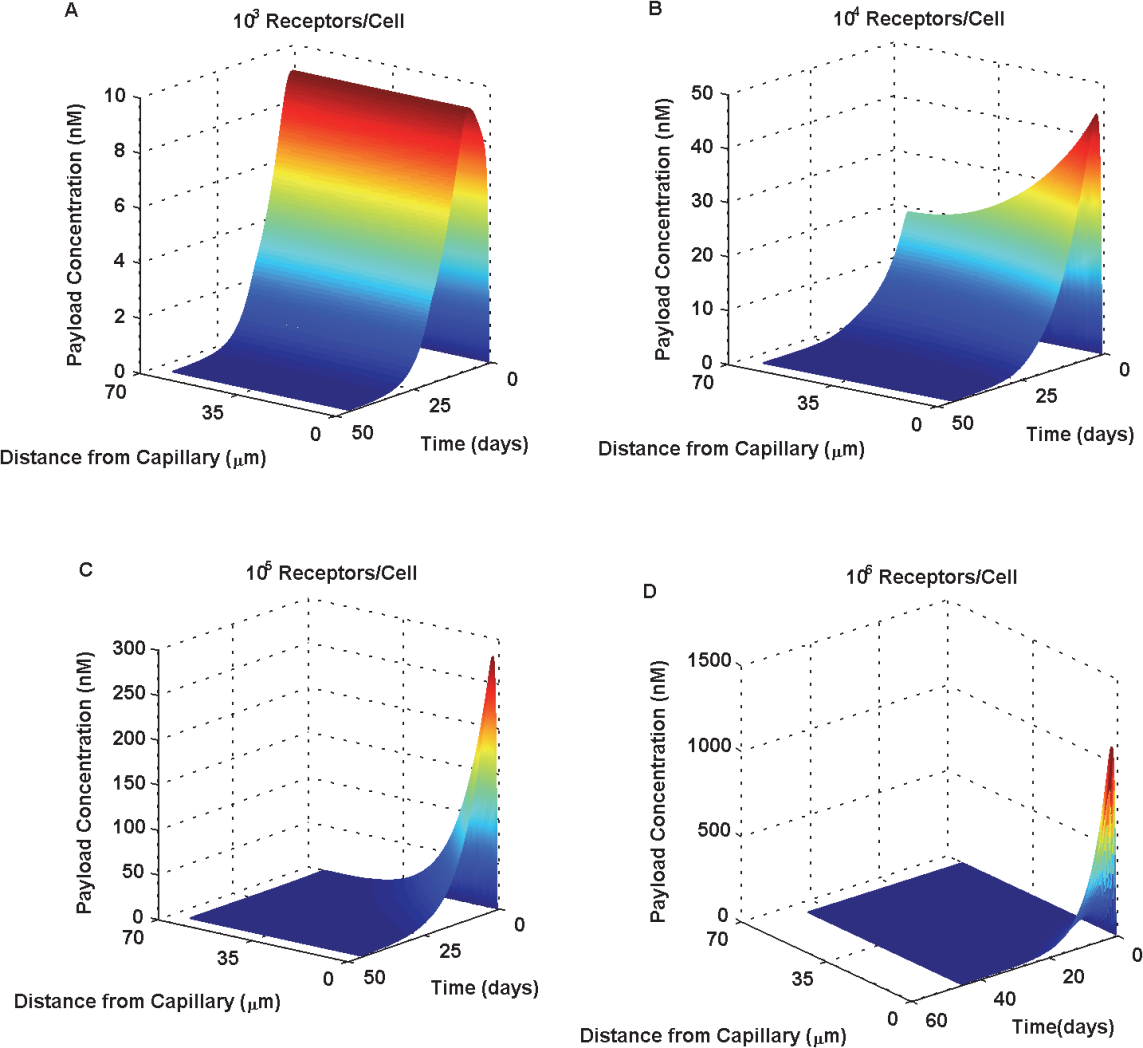
Increasing target receptor density has a profound effect on payload distribution across the tumor tissue. Receptors numbers per cell were varied from 10^3^ (A) to 10^4^ (B), 10^5^ (C) and 10^6^ (D). Low antigen (receptor) levels (A,B) resulted in more homogeneous payload distributions, as compared to high antigen levels (C,D) which produced pronounced, steep radial gradients. Increased receptor expression resulted in the rapid binding of ADC near its site of entry, an observation often termed as the “binding site barrier”.

To determine the effect of target receptor density on payload distribution across tumor tissue, the number of receptors per cell was first varied. Decreased antigen (receptor) levels were seen to produce more uniform, deeper-into-tissue concentration profiles (Fig. 2A), in contrast to higher antigen levels producing very steep radial gradients (Fig. 2C-D). Tissue heterogeneity observed for higher target expression was attributed to the rapid binding and immobilization of ADC near its site of entry, prohibiting the diffusion of the drug further away from the blood vessel; the so-called binding site barrier effect [30].

**Fig 3:**
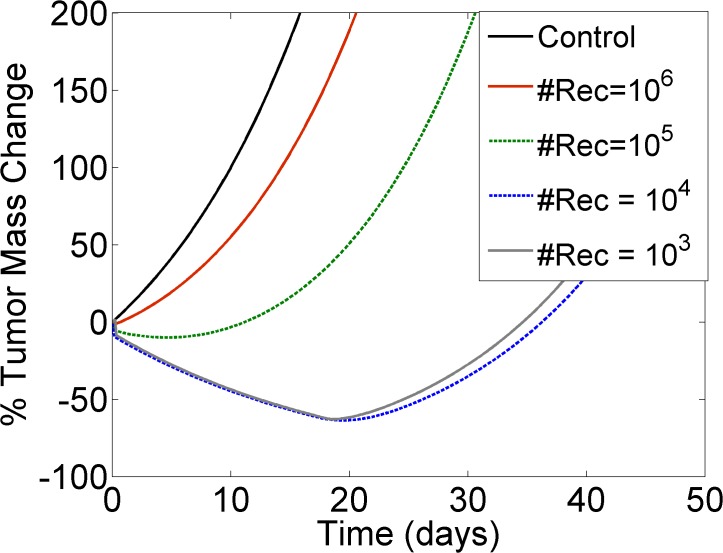
Tumor response to ADC therapy is driven by antigen expression levels. The Y-axis illustrates the % change in tumor mass from its initial value at time zero: negative values indicate a decrease and positive values indicate an increase in tumor mass from its baseline. Higher receptor numbers per cell (*#Rec*) diminish the ability of the tumor to shrink in response to ADC. Control (black solid line) was compared against increasing receptor densities: *#Rec* = 10^6^(red solid line), *#Rec* = 10^5^(green dashed line), *#Rec* = 10^4^ (blue dashed line) and *#Rec* = 10^3^(gray solid line). All simulations assumed a single intravenous administration of 1mg/Kg ADC, characterized by a binding affinity of K_D_ = 0.1 nM.

Target receptor levels were also shown to have an effect on the dynamic fluctuation of tumor mass over time (Fig. 3), as observed from the largely varied responses produced by a single intravenous administration of ADC. Tumors expressing low antigen levels were shown to reduce in mass, reaching a minimum after several days. In fact, lower receptor densities resulted in more prolonged and pronounced tumor mass reductions over time. Tumors with high receptor expression—in the order of 10^6^ receptors per cell—were unable to achieve any tumor mass reduction under the particular dosing regimen tested *(i*.*e*., only positive % tumor mass change values were observed).

**Fig 4:**
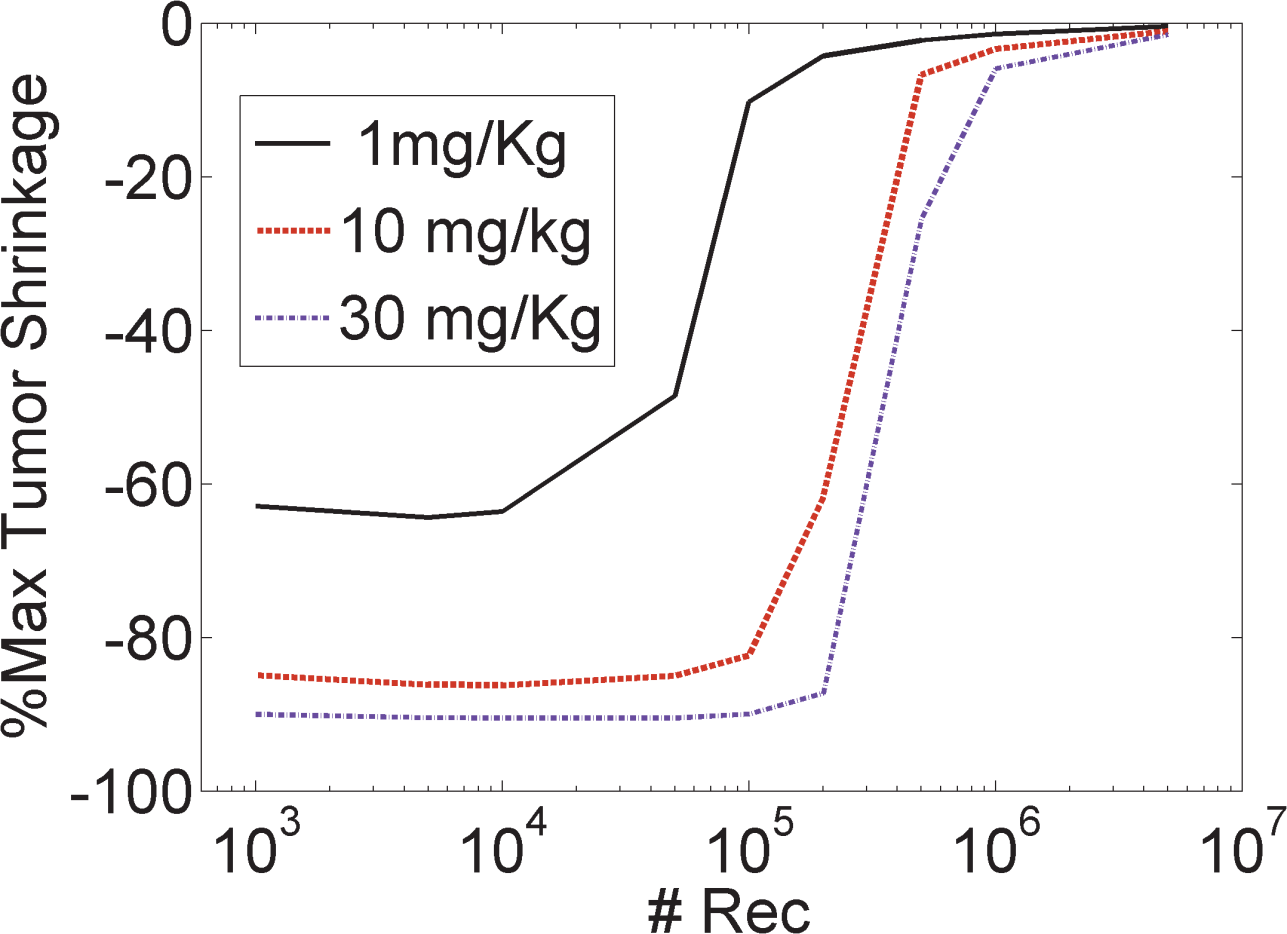
The maximum extent of tumor shrinkage as a function of antigen receptor levels. The Y-axis illustrates the maximal achievable tumor mass reduction; the more negative the values, the greater the tumor shrinkage, whereas a value of zero indicates that the tumor could not be shrunk. Lower receptor numbers (*#Rec*) produced improved tumor mass loss compared to higher receptor levels. Simulations assumed a single intravenous administration of either 1mg/Kg (black solid line), 10 mg/Kg (red dashed line) or 30 mg/Kg (purple dashed line) of ADC, characterized by a binding affinity of K_D_ = 0.1 nM.

To better visualize the effect of antigen expression on tumor mass shrinkage, we calculated the maximum tumor mass reduction *versus* the number of receptors per cell (Fig. 4). Tumors expressing lower receptor levels were easier to target and to reduce in mass, as compared to tumors expressing higher receptor levels. Increasing the number of receptors per cell above the 10^5^–10^6^ threshold resulted in the rapid impairment of ADC effect on tumor shrinkage. These results demonstrated that a decreased receptor density may, in fact, be a favorable property of the target system, implicating payload homogeneity in tumor tissues as an essential component to achieve effective ADC therapy.

**Fig 5:**
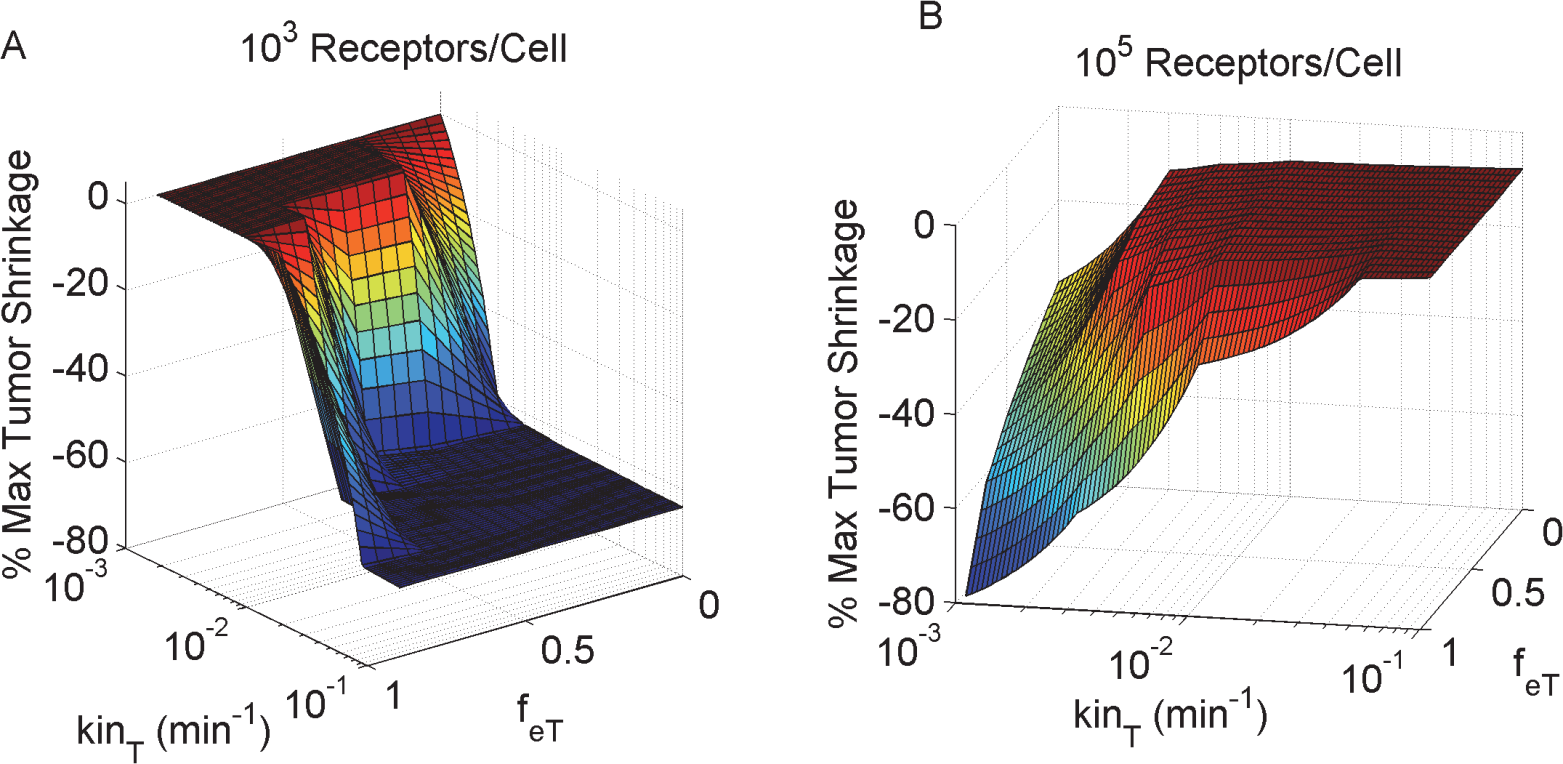
Tumor mass shrinkage as a function of target receptor kinetics. Increasing the internalization rate (*kin*_*T*_) and reducing the receptor recycling fraction (*f*_*eT*_) had a positive effect on tumor mass reduction, in the instance of low antigen expression levels (simulations of 10^3^ receptors per cell; A). The opposite effect is noted for tumors with high antigen expression (10^5^ receptors per cell B).

Next, receptor internalization rate and recycling fraction were simultaneously varied, to investigate their combined effect on the maximum tumor mass shrinkage. Tumors expressing low receptor levels were shown to benefit from more rapid receptor internalization rates and reduced recycling fractions (Fig. 5A). By contrast, tumors with increased receptor levels were seen to benefit from slower internalization rates and higher internalization fractions (Fig. 5B). This model output is in agreement with our previous findings which demonstrated that increased receptor binding immobilizes the antibody close to its site of entry, leading to reduced antibody penetration. Increasing the recycling fraction and reducing the receptor internalization rate allow for more antibody to penetrate further away from the capillary wall, thereby improving tumor mass reduction.

#### Tumor Xenograft Properties

**Fig 6:**
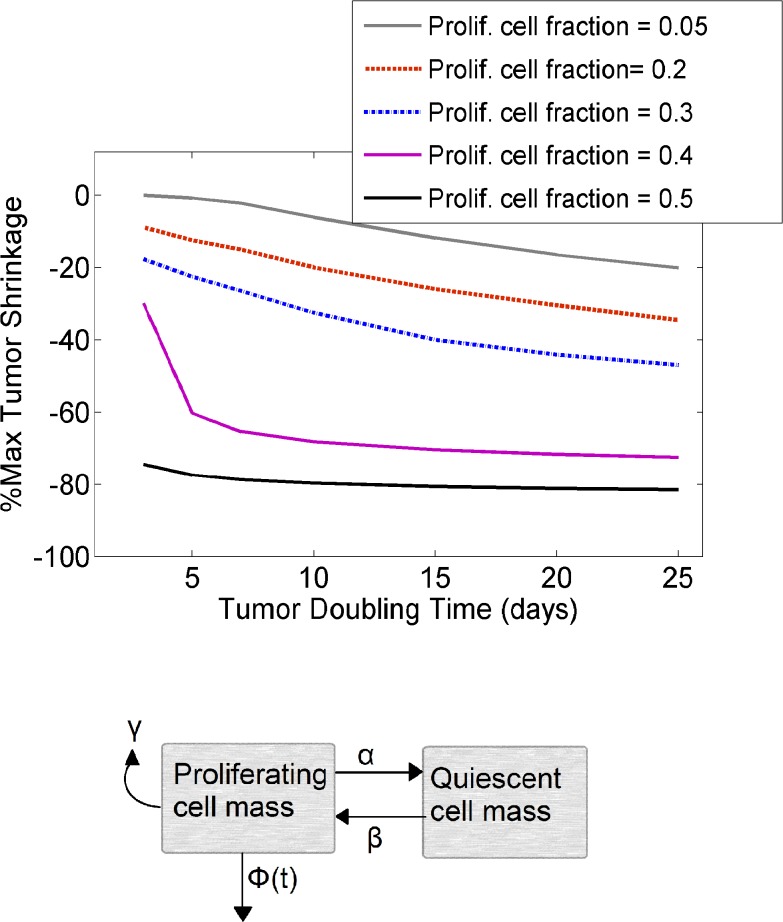
Intrinsic tumor growth properties affect the maximum extent of tumor shrinkage. A) Increasing tumor doubling time as well as the proliferating cell fraction enhanced tumor reduction in response to a single intravenous administration of 1mg/Kg ADC. The binding affinity was set equal to 0.1nM. Proliferating cell fractions simulated: 0.05 (gray solid line), 0.2 (red dashed line), 0.3 (blue dashed line), 0.4 (purple solid line) and 0.5 (black solid line). B) Simplified illustration of the tumor mass model, which included the dynamics of both proliferating as well as quiescent cell mass. Φ(t) indicates the proliferating mass reduction due to drug effect. More details can be found in the text.

Tumor doubling time (*t*_*d*_) and the proliferating-to-quiescent cell fraction (*r*_*t*_) were next varied, to investigate their combined effect on the maximum extent of tumor death. Note that tumor doubling time (*t*_*d*_), widely used as a quantification of tumor growth rate [40], is not equivalent to the doubling time of individual proliferating cells as part of the cell cycle (defined by *γ* in *Eq*. 15). Our simulations demonstrated improved tumor shrinkage with increased tumor doubling time and increased proliferating cell fractions (Fig. 6). The proliferating-to-quiescent cell ratio was shown to have a more pronounced effect on tumor mass reduction, as compared to the tumor doubling time.

**Fig 7:**
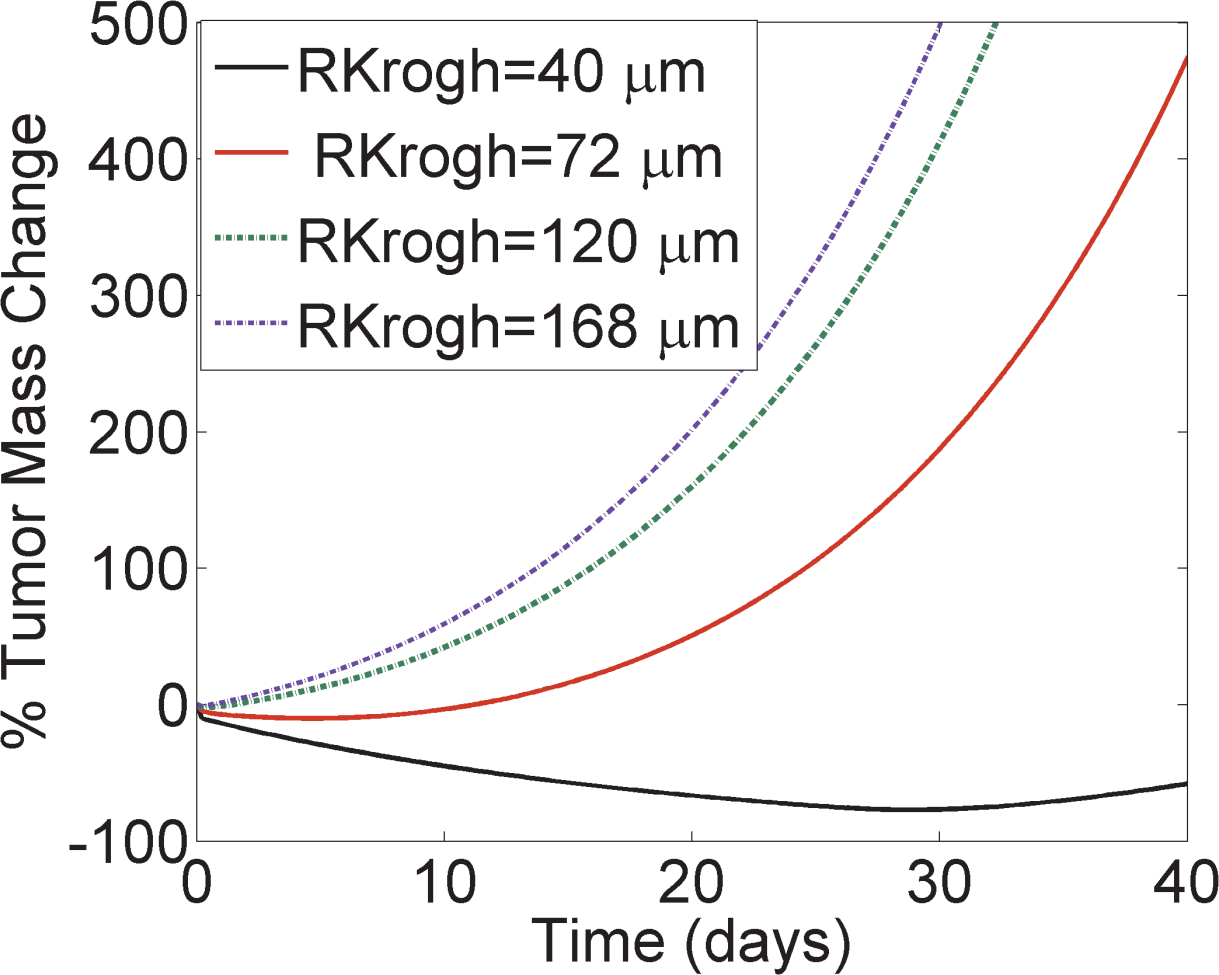
Tumor vascularization affects tumor response to ADC therapy. Increasing the Krogh cylinder radius (*RKrogh*) simulates tumors of decreased vascularization. *RKrogh* was increased from 40μm (black solid line), to 72 μm (red solid line), 120 μm (green dashed line), and up to 168 μm (purple dashed line). The graph demonstrates that highly vascularized tumors can reduce more promptly in contrast to less vascularized tumors, when applying the same ADC therapy. Dosing regimen simulated: single intravenous administration of 1mg/Kg ADC, characterized by a binding affinity of K_D_ = 0.1 nM.

To investigate the effect of tumor vascularization on tumor mass reduction, the Krogh cylinder radius was varied—essentially, a measure of the mean intercapillary distance (Fig. 7). Reduced *RKrogh* values produced tumors that were more accessible to the drug and therefore easier to target, as compared to less vascularized tumors characterized by high *RKrogh* values. Our simulations demonstrated that highly vascularized tumors reduce more promptly compared to less vascularized tumors, while applying the same dosing regimen. These modeling results suggest that xenograft selection plays a key role in study endpoints, indicating the need to interpret the success or failure of a compound in close relation to the intrinsic properties of the tumor.

### Extrinsic (ADC) Properties

**Fig 8:**
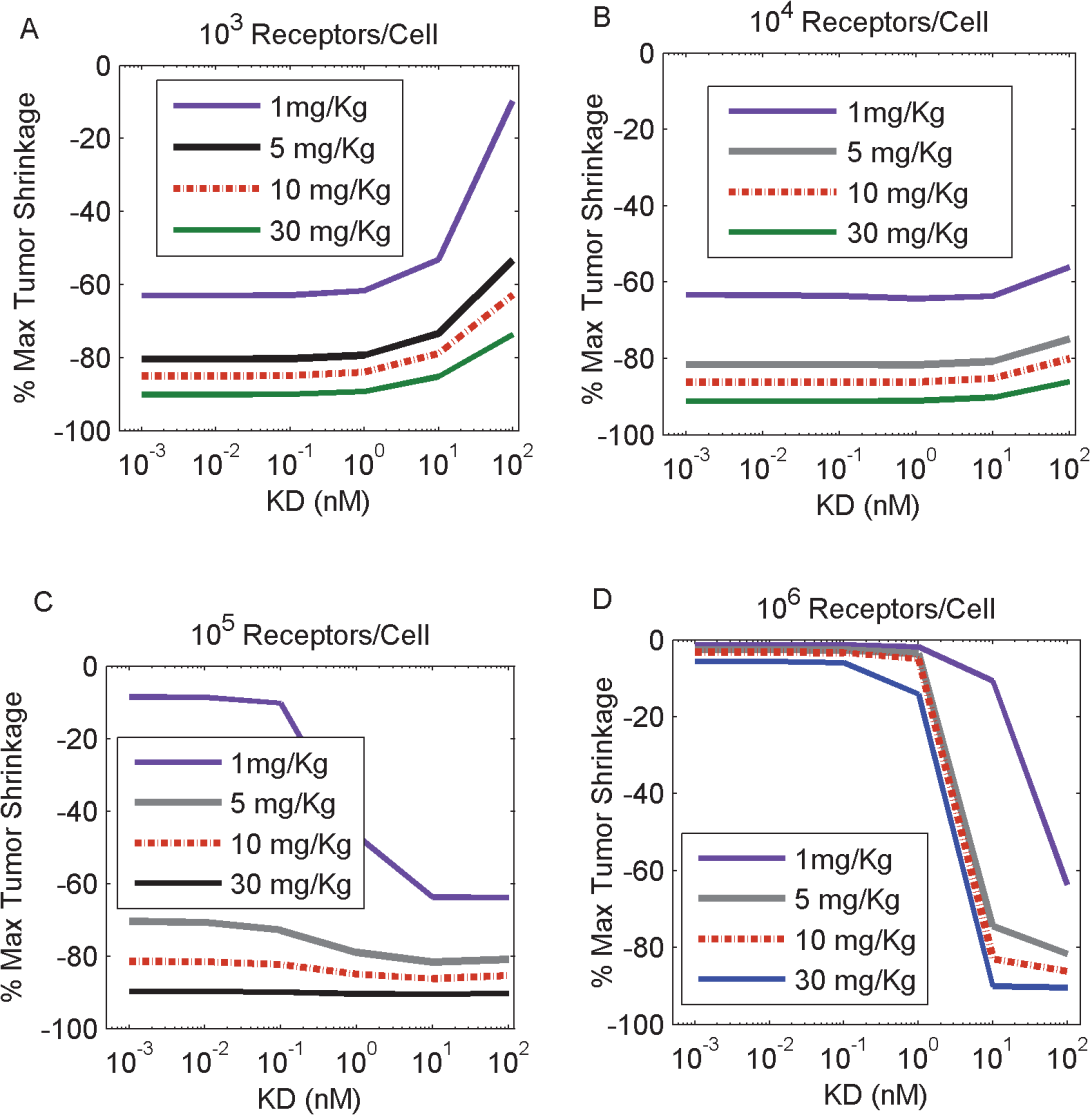
Increasing K_D_ produces a differential effect on tumor mass shrinkage, depending on antigen (receptor) level expression. Simulations included tumors that contained 10^3^ (A), 10^4^ (B), 10^5^ (C) and 10^6^ (D) receptors per cell. Increasing doses of 1mg/Kg (purple line), 5 mg/Kg (gray line), 10 mg/Kg (red dashed line) and 30 mg/kg (black line) of a single ADC intravenous administration were simulated. Our results show that decreasing K_D_ is beneficial when targeting tumors of low antigen expression. Contrarily, high antigen expressing tumors require increased K_D_ values to reduce in mass.

We next investigated the therapeutic potential of extrinsic drug properties, by varying the binding affinity and dose of administered ADC and determining their combined effect on tumors which expressed varying degrees of antigen levels (Fig. 8). Tumors expressing low antigen levels benefited, in a therapeutic sense, from low K_D_ values, as increasing the K_D_ monotonically produced inferior tumor mass shrinkage (Fig. 8A-B). By contrast, tumors with increased antigen levels exhibited an inverse response to high K_D_ values. In the instance of high receptor density, tumor death improved with increasing K_D_ and reached an asymptote after a K_D_ threshold value was achieved (Fig. 8C-D). As expected, higher doses of ADC produced improved tumor shrinkage for all tumor types, regardless of their antigen expression. These simulation results established a correlation between the intrinsic properties of the target tumor and the extrinsic ADC design optimization process.

To investigate the role of payload kinetics in terms of drug efficacy, scenarios of endosomal *versus* lysosomal payload releases were explored with the model, in addition to simulating the effect of increasing the payload retention half-life, *t*_*p*_. Endosomal payload release was simulated by modifying *Eq*.10 as follows:

$$\frac{d[C_{p}]}{dt}\;=\;k_{inB}\;B*DAR-k_{out}\left[C_{p}\right]+k_{in}\frac{\left[C_{p,ext}\right]}{\varepsilon }$$ 23

**Fig 9:**
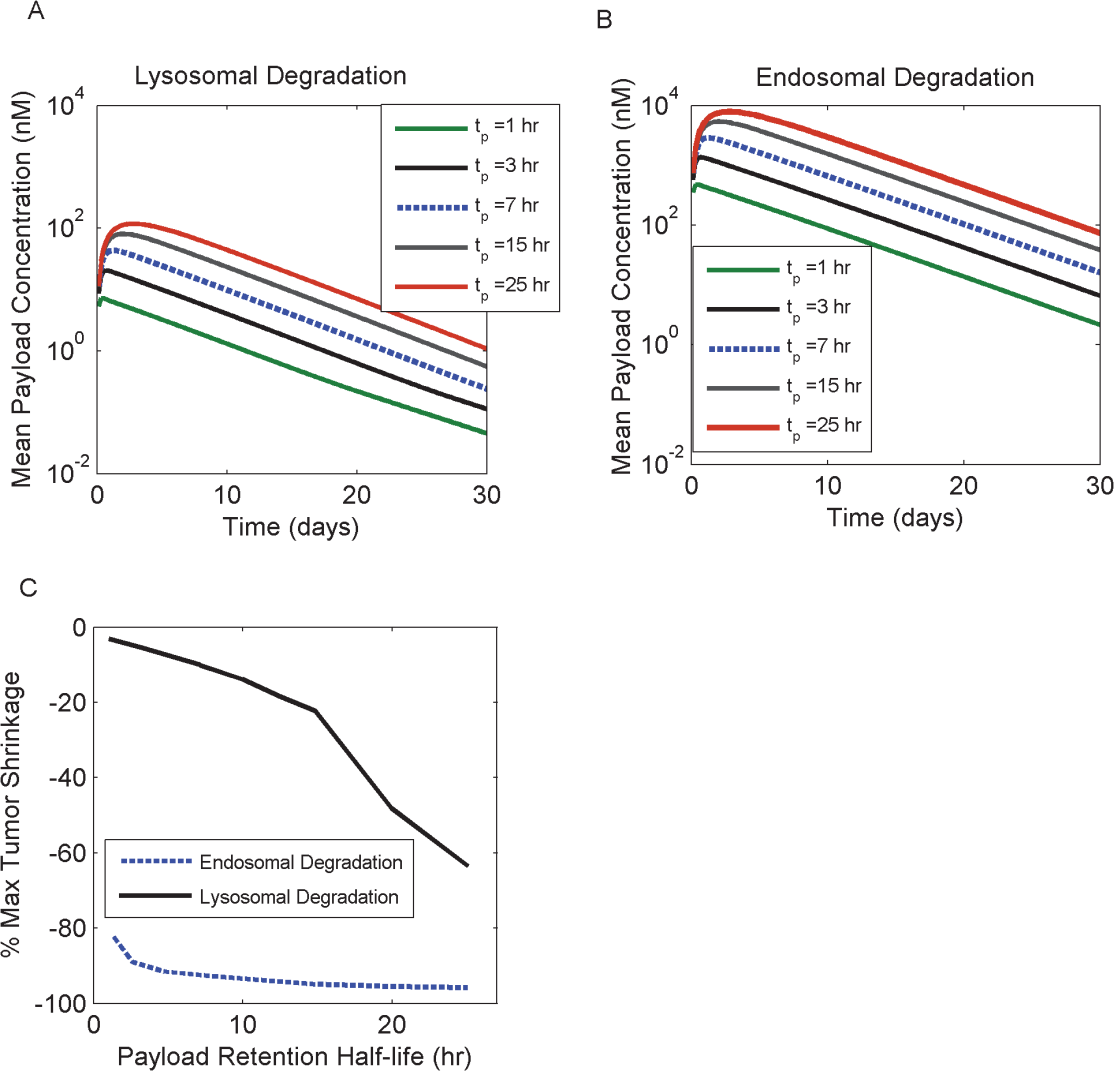
ADC efficacy as a function of payload cleavage mechanisms and intracellular kinetics. Lysosomal (A) *versus* endosomal (B) payload cleavage scenarios were simulated; the effect of payload retention half-life(*t*_*p*_) was also explored, by increasing it from 1 hr (green line), to 3 hrs (gray solid line), 7 hrs (blue dashed line), 15 hrs (gray solid line), and 25 hrs (red solid line). The maximum extent of tumor shrinkage (C) was improved for endosomal (blue dashed line) *versus* lysosomal (black line) payload cleavage and for longer retention half-lives.

Endosomal release resulted in much higher payload concentrations in the cytosol, as compared to lysosomal cleavage (Fig. 9A-B). Additionally, a positive correlation was observed between increasing the retention half-life, indicative of the payload transfer rate from the cytosol towards the extracellular space, and the payload accumulation within the cytosol. As a consequence, tumor mass suppression was more profound in the case of endosomal *versus* lysosomal payload release, and with increasing retention half-lives (Fig. 9C).

**Fig 10:**
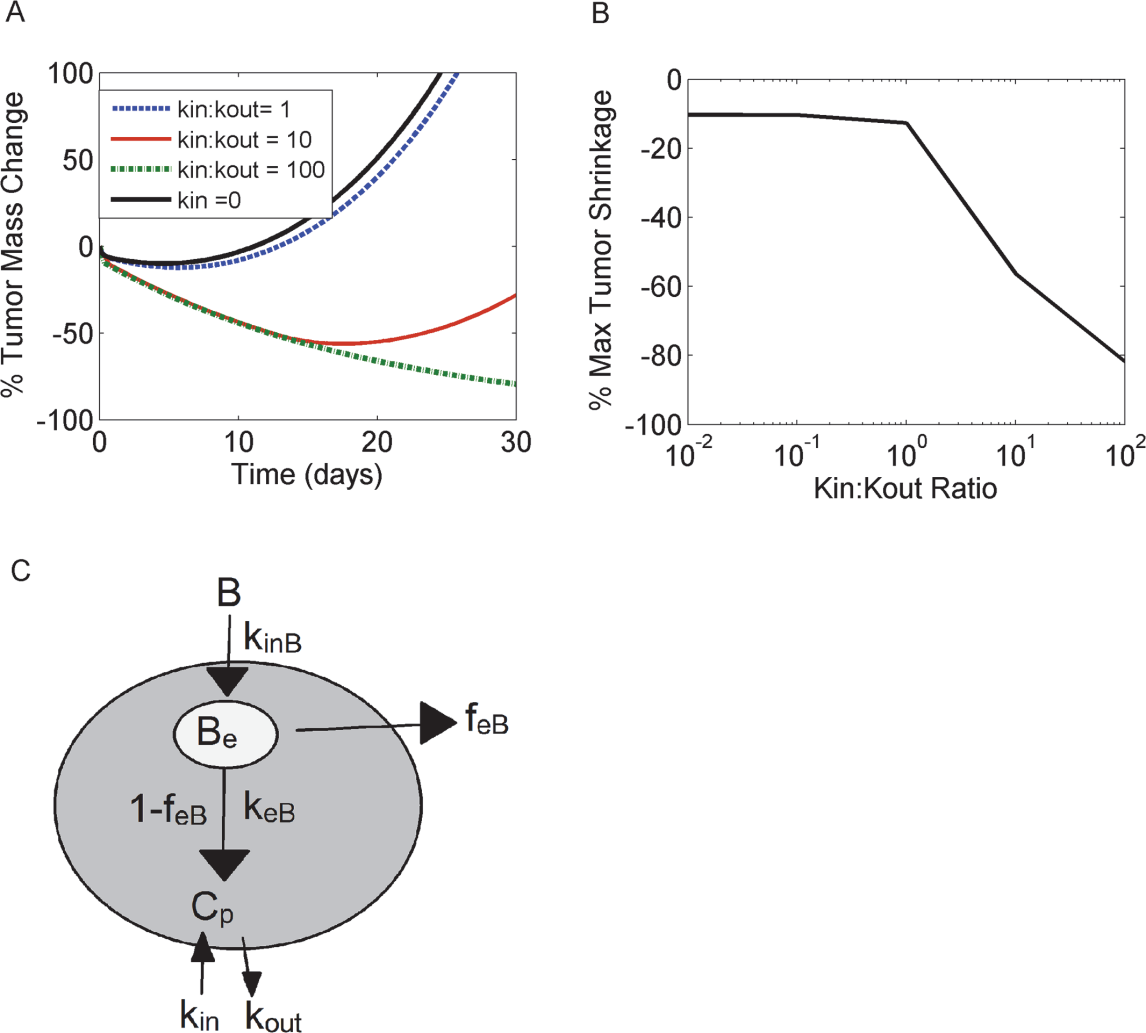
Bystander killing effects become significant depending on payload kinetics. Ratio of *k*_*in*_ (transfer rate from interstitial tumor tissue into the cytosol) *versus k*_*out*_ (transfer rate from the cytosol into the interstitial tumor tissue) was increased, and the effect on the % tumor mass change over time (A) and maximum tumor shrinkage (B) was reported. *K*_*in*_ with a nominal value of zero (black solid line), was gradually increased for the purpose of these simulations, to achieve *k*_*in*_-to-*k*_*out*_ ratios equal to 1 (blue dashed line), 10 (red solid line) and 100 (green dashed line). C) Simplified illustration of ADC kinetics and payload release. Payload accumulation within the cytosol depends on *k*_*in*_ and *k*_*out*_ transfer rates. For more information on intracellular kinetic equations, refer to text.

We also addressed the question of bystander killing effect and investigated payload properties that can cause cytotoxicity to neighboring cells. For these simulations, payload re-entry into the cytosol from the local tumor environment was allowed in the model, and the ratio of transfer rates between interstitial tumor tissue and cytosol was gradually increased (kin/kout; Fig. 10A). Of note, these simulations did not include the release of payload upon cell death and lysis into the interstitial space. Simulations showed that tumor cell death due to bystander effects became significant only when the payload re-entry rate (*kin*) became greater than the exit rate of the payload from the cytosol (*kout*). A steep decline in tumor mass was observed when *kin/kout* >1, indicative of an onset of significant bystander killing effects (Fig. 10B).

## Discussion

We developed a generalized mathematical representation of a solid tumor in a mouse model, incorporating essential mechanisms involved in ADC tumor localization and distribution. Our focus was on both intrinsic properties of the tumor itself, as well as controllable design parameters to further our understanding on effective drug delivery and ADC drug design optimization.

Our simulations demonstrated a largely varied tumor response to ADC therapy, depending on target receptor properties. It is these varied tumor responses that will guide the design of successful therapeutic agents, under conditions encountered in clinical pathology. For the purpose of this study, we varied receptor expression levels and endocytic trafficking dynamics. Receptor properties (such as K_D_, recycling rate, internalization rate and receptor expression number) were shown to be primary drivers of payload distribution profiles across the tumor tissue: with more uniform distributions being more beneficial, in contrast to more heterogeneous, steep gradient profiles, indicative of reduced tumor penetration. Drug concentration gradients (Fig. 2 C-D) can be interpreted as a competition between local ADC/receptor binding and metabolism *versus* diffusion of the drug in the tissue. When diffusion is more rapid than immobilization, not-as-steep radial gradients are observed. Therefore, as target receptor expression levels increase, diffusive limitations become more prominent and cellular tissue further away from the vessel becomes inaccessible. This concept, proposed more than 20 years ago, is termed as the “binding site barrier” [30]. Experimental studies focusing on the role of increased target density and receptor turnover rates have consistently provided growing evidence in support of this theory [41, 42].

We next focused on strategies that would be expected to increase payload tissue homogeneity, which is correlated with enhanced ADC efficacy. In particular, we investigated the role of antibody binding affinity. Higher K_D_s allow for a more “loose” binding state between ADC and target, enabling the drug to reach more distant targets. Similar findings have also been reported experimentally, for both antibodies as well as antibody fragments (such as single chain variable fragments, scFvs), further supporting the correlation between inefficient tumor penetration and too high affinities [43, 44]. Proper choice of antibody affinity can therefore balance unfavorable system properties such as high antigen expression, rapid internalization and turnover, which would otherwise limit the therapeutic effects of the drug. Hence the model supplies a quantitative solution to the binding site barrier dilemma.

A key design feature of ADCs is the linker connecting the cytotoxic agent to the antibody. Linker stability can influence the site and amount of payload cleavage along the intracellular trafficking pathway—with some linkers permitting the release of the cytoxic agent in earlier endosomal compartments, bypassing the need for trafficking to the lysosomes [45, 46]. Additionally, linker cleavage produces a range of metabolites whose properties, such as size and lipophilicity, are closely associated with the chemical nature of the linker used [35]. Metabolite properties directly affect payload efflux rates from the cytosol as well as payload reentry into the cell. As a consequence linker design is a key determinant of *in vivo* efficacy [47, 48]. Payload reentry has, in fact, received increasing interest due to its implication in bystander cell killing effects and its ability to enhance ADC efficacy [47, 49]. In this work, we systematically varied payload kinetics to mimic the effects of linker selection. In our simulations, increasing payload retention half-lives were associated with improved tumor shrinkage, reflecting the impact of ADC metabolites and their kinetic properties in drug efficacy. Exploration of the competing scenarios of lysosomal *versus* endosomal payload release demonstrated superiority of the endosomal cleavage mechanism, which tends to increase the cell-killing potency of the ADC. It should be noted, however, that lysosome-independent mechanisms are characteristic of “cleavable” linkers, which are known to produce free payload or simple derivatives intracellularly, but are also less stable in circulation [46]. The advantage of endosomal cleavage is therefore tempered by lower systemic stability (*i*.*e*., shorter ADC half-lives). This may reduce ADC tumor exposure and concomitantly result in toxicity in normal tissues [46]. Our model has not explored reduced ADC exposures associated with “cleavable” linkers; rather we have assumed one common PK profile regardless of the linker utilized. Finally, we looked into bystander killing effects and the conditions which allow neighboring cell cytotoxicity. Our model demonstrated that the payload uptake rate in the cancer cell needs to be higher than the payload efflux rate out of the cancer cell, in order to effectively observe bystander effects. These calculations agree with the recent modeling work on bretuximab vendotin from Shah *et al*. [11], who computed a ratio of kin:kout = 10:1.

This is the first example of a quantitative model that integrates key mechanistic features of tumor penetration, ADC distribution dynamics and patho-bio-physiology. This work was built on a previous model which describes vascular permeability and diffusion kinetics [7], to incorporate additional system components, such as endocytic trafficking mechanisms, payload release and tumor mass dynamics. The complete tumor penetration model allows for a thorough exploration of multiple parameter combinations leading to a better understanding of success or failure scenarios of ADC therapeutics. For instance, the model predicts reduced therapeutic effect against tumors expressing high levels of receptors, also characterized by fast internalization and low recycling fractions. But if the receptors within the same high-antigen-expressing tumor were to be internalized at slower rates, or recycled at higher fractions, a much improved therapeutic effect would be expected. Interestingly, this scenario is similar to TDM1, which targets Her2 receptors. Her2 expression reaches approximately 2*10^6^ receptors per cell but also exhibits a highly increased recycling fraction of ∼0.95 [50]. The binding site barrier effect which would be expected due to high antigen expression is therefore counteracted by the increased recycling fraction of the receptor. Using this modeling platform, we may therefore customize simulations to address questions specific to a particular tumor type or receptor. An extension to this work would be the integration of the current running model—descriptive of the tumor dynamics—with the ADC effect in the healthy tissue, to project toxicity issues and estimate therapeutic index.
